# Dual Kidney Transplantation: A Review of Past and Prospect for Future

**DOI:** 10.1155/2017/2693681

**Published:** 2017-07-02

**Authors:** Muhammad Abdul Mabood Khalil, Jackson Tan, Taqi F. Toufeeq Khan, Muhammad Ashhad Ullah Khalil, Rabeea Azmat

**Affiliations:** ^1^Diaverum Prince Abdul Majeed Renal Centre, Al Imam Ahmad ibn Hanbal, Jeddah 21146, Saudi Arabia; ^2^RIPAS Hospital, Bandar Seri Begawan BA1710, Brunei Darussalam; ^3^King Salman Armed Forces Hospital, TabukKing Abdul Aziz Rd., Tabuk 47512, Saudi Arabia; ^4^Khyber Teaching Hospital, Peshawar, Khyber Pakhtunkhwa 25000, Pakistan; ^5^Aga Khan University Hospital, Karachi 74800, Pakistan

## Abstract

Kidney transplantation (KT) is one of the treatment options for patients with chronic kidney disease. The number of patients waiting for kidney transplantation is growing day by day. Various strategies have been put in place to expand the donor pool. Extended criteria donors are now accepted more frequently. Increasing number of elderly donors with age > 60 years, history of diabetes or hypertension, and clinical proteinuria are accepted as donor. Dual kidney transplantation (DKT) is also more frequently done and experience with this technique is slowly building up. DKT not only helps to reduce the number of patients on waiting list but also limits unnecessary discard of viable organs. Surgical complications of DKT are comparable to single kidney transplantation (SKT). Patient and graft survivals are also promising. This review article provides a summary of evidence available in the literature.

## 1. Introduction

KT when compared with dialysis offers improved survival, better quality of life, better social rehabilitation, and less economic cost. The number of kidney transplantations is increasing worldwide. Similarly the number of patients waiting to get a kidney has also increased tremendously. The number of patients on waiting list for kidney in July 2016 was 99,413 in USA as per data of Organ Procurement and Transplantation Network (OPTN). Unfortunately only 17,878 patients could get a kidney [1]. Despite recent relaxation of rules to accept expanded criteria donors (ECD), the gap between demand and supply is still huge. Around 20 to 40% of ECD and dual kidneys recovered were discarded in US [2]. The discard rate is 8% in Europe which is comparatively lower than the United States of America [3–5]. Theoretically, increased nephron mass supply by simultaneously transplanting two suboptimal kidneys to the same recipient may work better than a single kidney. This will both reduce the number of the patients on waiting lists and discard rates. In this review we will examine the current evidence available and discuss the concept of DKT, criteria for donor and recipient evaluation, surgical techniques and its complications, outcomes of DKT, and new prospects and future directions for DKT. This review does not include pediatric dual kidney donation or implantation.

## 2. Concept of Dual Kidney Transplant

Adequate nephron mass is a predictor of long term graft outcome. The nephron dose concept is a known terminology to transplant physicians. Nephron dose concept conveys that any reduction in nephron mass causes hyperfiltration and hemodynamically mediated glomerular injury [6]. The theory of hyperfiltration related injury is well known in transplant nephrology. It has been shown to be associated with reduced graft survival, when the kidney-to-recipient weight ratio is below 2.0 g/kg [7]. Animal studies have shown that sufficient nephron mass by doing DKT when compared to SKT prevented long term deterioration in kidney functions [8, 9]. Higher nephron mass in humans through DKT should theoretically reduce the deterioration in long term graft function [10]. Logically, single kidney from ECD has less number of functional nephrons when compared with two ECD kidneys which should translate to better overall kidney function. Organ preservation, ischemia reperfusion injury, exposure to calcineurin inhibitors, rejections, and hypertension in posttransplantation period have deleterious effects on renal parenchyma. Single kidney from ECD by virtue of having less functional renal parenchyma will be more vulnerable to damage by these factors.

## 3. Criteria for Selection of Donor

Criteria for dual kidney transplantation are highly variable among centers across the world. The decision for DKT has to be taken with great caution and deliberation. The practice of discarding kidneys from ECD has generated many discussions about wastages and prodigality, especially in the current climate where transplant waiting lists are ever increasing. There is now plenty of evidence that DKT can achieve good long term outcomes, often comparable to SKT. On the other hand, putting dual kidneys with inadequate nephron mass may be hazardous because some evidence has shown that patients who require dialysis after failed marginal grafts had higher mortality and morbidity compared to those without a history of kidney transplantation [11, 12]. Keeping these pros and cons in mind, we feel that a meticulous scrutiny of criteria is needed to ensure that recipients achieved desirable outcomes. Unfortunately the criteria for selection are highly variable among various transplant centers and there is no universal consensus on the best way forward.

ECD is defined as all deceased donors ≥ 60 years of age or donors who were 50–59 years of age and had two of the following: donor history of hypertension; donor death due to cerebrovascular accident/stroke; or terminal serum creatinine value greater than 1.5 mg/dl. ECD needs meticulous evaluation before deciding to do SKT or DKT. Since the start of DKT in 1996 to 2013, various selection criteria were proposed and utilized. Various criteria are considered: age, presence of comorbidity (diabetes or hypertension), cold ischemia time, creatinine clearance, and preimplantation biopsy finding for allocation. Preimplantation biopsy finding predicts long term outcome of the graft. Karpinski et al. found that donor vessel scores can predict delayed graft function and graft survival after transplantation [13]. Using Banff criteria in preimplantation biopsy and doing combined evaluation of donor glomerulosclerosis, chronic vascular and interstitial damage allows a precise prediction of graft outcome [14]. Various other studies used biopsy scores to decide to opt for DKT or SKT [15, 16].

Johnson et al. in 1996 did preimplantation biopsy and did DKT in donors having less than 40% glomerulosclerosis without severe interstitial fibrosis or arteriosclerosis on biopsy [15]. Beside biopsy they considered age, comorbidity (diabetes/hypertension), and cold ischemia time and creatinine clearance. They included donors with cold ischemia time less than 30 hours and with creatinine clearance levels between 40 and 80 mL/min. Using histology together with other donor factors their criteria for selection of donor led to 100% survival at 6 months.

Remuzzi et al. [16] assessed their biopsies and used scores for glomerulosclerosis, tubular atrophy, interstitial fibrosis, and arterial and arteriolar narrowing. They graded each element from 0 to +3, with a total maximal score of 12. The final grade was labelled mild if the score was 0–3, moderate 4–6, and severe 7–12. The kidney with mild grade label was allocated for SKT and moderate grade (4–6) for DKT.

The kidneys with severe grade (7–12) were discarded. Beside biopsy they also considered age, comorbidity, and proteinuria while selecting donors for DKT. Despite slight differences from Johnson et al. criteria the graft survival reported by Remuzzi et al. was 100% at 6 months and 93% at 3 years.

Preimplantation biopsy also helps in selection of donors across a wide range of donors irrespective of their age. Using age and histological finding together can help in sensible allocation for SKT or DKT with reasonable outcome irrespective of age limit. Andrés and his colleagues [17] selected cadaveric donors with normal creatinine irrespective of age limits and performed pregraft biopsy in donors with age greater than 60 years to assess glomerulosclerosis. A DKT was done when the donor age was 75 years or older or when the donors between 60 and 74 years old and had a glomerulosclerosis of more than 15%. Using this selection criterion the graft survival was 95% graft survival at 1 year and 93% at 2 years. Promising graft survival was reported despite the fact that their cohort received kidneys from very elderly population.

In a data review of UNOS kidney biopsy along with other parameters was used to select donors for DKT [18]. Preimplantation glomerulosclerosis between 15% and 50% was one of the tools for selecting donors. Parameters considered for selection included age greater than 60 years, creatinine clearance greater than 65 mL/min, rising serum creatinine greater than 2.5 mg/dL at retrieval, chronic hypertension or type 2 diabetes mellitus, and glomerulosclerosis on biopsy between 15% and 50%. DKT was done if any of the two parameters was present. The selection criterion was reasonable as translated by 5-year patient survival 95.6% in their cohort.

Yet in another study kidney biopsy was restricted for allocation in high risk ECD [19] for allocation. Criteria used for high risk ECD included elderly donor with age ≥ 70 or 60–69 with one of the following risk factors:

- Serum creatinine > 1.5 mg/dl.
- Calculated creatinine clearance ≤ 60 ml/minute.
- History of hypertension and/or diabetes.
- Proteinuria > than 1 gram.
- Cause of death cerebrovascular accident.

They did kidney biopsy in high risk marginal donor and assessed biopsies using Karpinski and Remuzzi histological scores. SKT was done when score was 0–3. Kidneys with score 4–6 were allocated for DKT and those with score 7–12 were discarded. The group demonstrated that graft ad patient were similar to SKT.

Some centers used kidneys for DKT refused by other local centers for a variety of reasons. One of the reasons was suboptimal pretransplantation biopsy. This subgroup underwent DKT and was studied by Lu et al. [20]. Reasons for refusal were multiple including history of hypertension, donor instability, donor age, or marked elevation in donor creatinine level after hospital admission, suboptimal pretransplantation biopsy findings, or a combination of these factors. Lu et al. found that recipients of DKT from these ECDs have excellent outcomes. The good outcome in this data was promising despite the fact that recipients of double kidneys were older and had a lower creatinine clearance on hospital admission. Other authors also used biopsy along with various clinical parameters for selection of the donor [21, 22].

From reviewing the evidence so far discussed it is evident that biopsy before transplantation was a vital selection tool. However, work from other colleagues used selection criteria not using preimplantation biopsy. Interestingly parameters from hypothermic machine perfusion with measurement of enzymes for ischemic injury have been used in donation for DKT after cardiac death. Donation after cardiac death (nonheart beating) is considered ECD because of long warm ischemia time. Navarro et al. [23] used hypothermic machine perfusion to preserves the organs. They assessed pressure flow index (defined as flow per 100 grams renal mass divided by systolic blood pressure) and concentration of glutathione transferase, an enzyme marker of ischemic injury. SKT was done when pressure flow index was 0.4 mL/min per 100 g/mm Hg and glutathione transferase was less than 100 IU/L/100 grams renal mass. Kidneys were discarded if pressure flow index was less than 0.4. DKT was done when if pressure flow index was satisfactory but glutathione transferase was higher than the cut-off value. Patients having comorbidities and prolonged cold ischaemia also underwent DKT. The group concluded that viability testing in nonheart beating donors can help in distinguishing kidneys that may be unsuitable for SKT but when used as double transplant have the potential to produce sufficient renal function.

Similarly glomerular filtration rate (GFR) alone in elderly patients was used in allocation of the kidneys either for DKT or SKT without doing a kidney biopsy before implantation. Snanoudj and his colleagues [24] prospectively compared DKT and SKT receiving grafts from ECD donors aged > 65 years and allocated kidneys according to donor estimated glomerular filtration rate. DKT was done if estimated glomerular filtration rate was between 30 and 60 mL/min and SKT if estimated glomerular filtration rate was greater than 60 mL/min. At the end of 12-month follow-up, GFR was similar between the two groups. The group then advocated the importance of GFR in allocation of kidney from elderly population without doing a biopsy. They argued that delaying the transplant to obtain histology will increase cold ischemia time. Moreover, emergency histopathological reporting is also an issue at various centers. There are also some critics who are of the opinion that kidney biopsy may lead to more discard [25].

Organ Procurement and Transplantation Network (OPTN) and United Network for Organ Sharing (UNOS) introduced KDRI (Kidney Donor Risk Index) and KDPI (Kidney Donor Profile Index) to quantify risk scores for deceased donor kidneys. The KDRI is an estimate of the relative risk of posttransplant kidney graft failure from a particular deceased donor compared to a reference healthy donor of age 40 years [26]. KDPI of higher than 80% predicts high risk of graft failure; however there is no cut-off for accepting or rejecting a kidney [27]. Therefore one must be cautious while taking decision on the basis of KDPI. However, Klair et al. used KDRI for DKT and concluded that KDRI > 2.2 is a useful discriminatory cut-off for the determination of graft survival [28].

It is cleared from discussion so far that many centers used histological tool along with various clinical parameter for allocation. Others relied on hypothermic perfusion parameters or estimation of GFR or kidney donor profile index (KDPI) without doing a biopsy. The preimplantation biopsy can have pitfalls. They may sample a zonal scar and may not be a true representation of the kidney. Superficial biopsies may not sample adequate arteries and arterioles; therefore the vasculature may not get evaluated. That is a significant disadvantage. Moreover, Shallow wedge biopsies can overestimate glomerulosclerosis, owing to the increased incidence of this in the subcapsular region [29]. The methodology for preparing the histologic sections of the preimplantation biopsy is also important. Frozen sections contain substantial freeze artifact, making interpretation difficult. Frozen sections are not reliable for assessment of mesangial cellularity, glomerular capillary wall thickening, some diabetic lesions, microthrombi, and acute tubular necrosis [30]. We advise rapid processing of formalin fixed permanent sections [30]. Lastly, it is also important for who reads this preimplantation biopsy. Not all pathologists are familiar with reading kidney pathology. All these factors are potential problems with preimplantation biopsies. Therefore, it is important to integrate histological scoring with clinical criteria and donor risk index. The aim should be to avoid discard and benefit greater number of patients waiting on the list. At the same time one should take care not to implant two kidneys where one kidney will be sufficient to provide optimal long term benefits. On the other side one should be careful not to implant kidneys with little reserves. This is due to fact that recipient with failed graft do worse latter on hemodialysis [11, 12].

**Figure 1:**
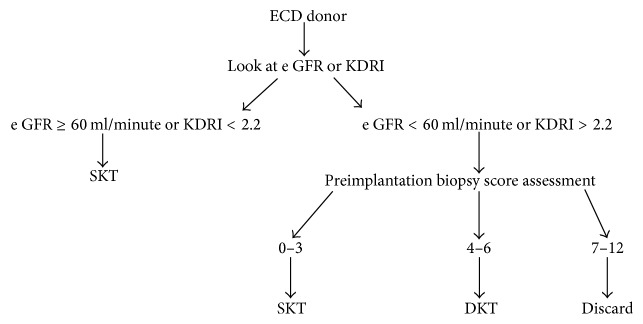


A reasonable way forward will be to estimate GFR or KDRI in all ECD. If e GFR is greater than 60 ml/minute or KDRI is less than 2.2 then kidneys should go for SKT [24, 28]. If e GFR is less than 60 ml/minute or KDRI is greater than 2.2 then these patients should undergo biopsy to decide for SKT versus DKT. The biopsy can be evaluated by Karpinski et al. or Remuzzi et al. histological scores [13, 16]. SKT should be done if score is 0–3. Kidneys with score 4–6 should be allocated for DKT and those with score 7–12 should be discarded.  Figure 1 showed the allocation scheme.

It will be nice to integrate histological score into multifactor score for selection of the donor to reduce discard and improve outcome. A consensus in transplant community for integrating various scores and coming up with selection criteria is also needed.  Table 1 summarizes the criteria for DKT.

## 4. Criteria for Selection of Recipient

Generally, the recipients of DKT were older when compared to SKT. Results of most studies showed that elderly patients who had DKT tend to have lower metabolic rate and low body mass index than the average SKT patients [16, 19, 20, 24, 28, 31, 32]. DKT is considered better for age and weight matched recipient. Greater number of nephrons in DKT is suitable for elderly patients with low basal metabolism and reduced body mass. The results of DKT in elderly were comparable with the younger SKT population [32].Theoretically elderly recipients tend to have blunted immunologic responses and, therefore, despite increased nephron mass, the chances of rejection are lower. Furthermore, there were promising results showing that DKT in a younger cohort (mean age 60 ± 5 years) from older donors (mean age 75 ± 7 years) had fewer episodes of acute rejection and good graft survival [17].

Bearing this evidence in mind, DKT should be offered to elderly patients with lower immunological risk and a normal body mass index. Younger patients may invariably have better outcomes but should be made aware that long term survivability of grafts may not match their life expectancy and may complicate their sensitization for future transplants.  Table 2 summarizes characteristics of the recipient who underwent DKT.

## 5. Surgical Technique for DKT

Various techniques are used for DKT including the extra- or intraperitoneal bilateral placement of the two kidneys [33–35] through two separate Gibson incisions or one midline incision [34–36]. Masson and Hefty were the first to transplant both adult donor kidneys unilaterally (monolateral or ipsilateral) into the same iliac fossa [37]. Their point of view was that there will be less trauma and less operative time is required to do the procedure. Furthermore, they argued that other side can be used for future transplantation if needed. However extensive dissection is needed in the later technique and there is a fear that this approach may be associated with renal vein thrombosis due to compression by dual kidney [19]. However Ekser et al. compared unilateral placement of dual kidneys and compared their results with SKT. They found the procedure safe and with good outcome [19].

Compared to SKT, dual anastomosis of the vessels and ureters is needed in DKT. Implantation of the two kidneys requires more dissection, surgical, and anesthetic time. This means that intraoperative medical and surgical complications are expected to be higher than SKT. Monolateral placement through a single Gibson incision reduces operating time significantly and is shown to be associated with lower surgical morbidity [28]. Some studies have suggested that bilateral DKT in recipients >60 years old due to longer period of anesthesia results in greater surgical risk [34, 38]. In view of these findings they suggested that DKT should be done in recipients of less than 60 years of age. However, Remuzzi et al. [16] who did bilateral placement of the kidneys reported that overall incidence of major surgical complications were comparable to SKT. Similarly, Esker and his colleagues [19] who did unilateral kidney placement found that there were no significant increases in the surgical or anesthetic complications in 60% of their cohort who were >60 years of age at the time of the transplant.

Renal vein thrombosis is a potentially serious complication that often leads to graft loss. The incidence is around 0.5 to 4% [39] in SKT. It is argued that unilateral DKT may be associated with renal vein thrombosis [19]. Ekser et al. in their cohort of unilateral DKT transplants showed that the incidence of renal vein thrombosis was 1% as compared to 5% in SKT [19]. Similar incidence of renal vein thrombosis was reported in other studies [24, 31]. Few other studies done in DKT did not show any renal vein thrombosis [16, 21, 40]. Similarly the incidence of arterial thrombosis was not significant between DKT and SKT [21, 24].

Lymphocele has been reported in 0.6–36% of SKT [41]. In DKT, the incidence of lymphocele has been reported as 3–15%. The occurrence of lymphocele was not statistically different between DKT and SKT in some studies [19, 21]. Even some studies done in DKT did not show any lymphocele [16, 18, 24]. Islam et al. [22] reported significant ureteral strictures in DKT. However other authors reported similar incidence of ureteral strictures between DKT versus SKT [19, 24]. In other studies no ureteral stricture was found [16, 21].  Table 3 summarizes complications of DKT reported by various authors.

DKT requires more time and the number of anastomosis doubles for the surgeon. However, with experience accumulating the complications associated with DKT are comparable with SKT. Keeping in mind good graft and patient survival in DKT and comparable complication rate with SKT, DKT will not only reduce discard of organ but also give an opportunity for recipients to live long and lead a healthy life.

## 6. Outcome of Dual Kidney Transplantation

Outcome of DKT transplantation can be measured by assessing various outcome variables. Like SKT, DKT can have delayed graft function and rejection. Long term outcome can be measured by looking into data for graft and patient survival.

Delayed graft function occurs in 10–31% cases in patients with DKT [16, 18–22, 24, 32, 34]. Snanoduj et al. [24] reported significantly less delayed graft function in DKT when compared with SKT (31.6% versus 51.4%) suggesting that DKT may be associated with less delayed graft function. However, this could not be reciprocated by various other studies [16, 18–22, 32, 34]. All these studies showed that delayed graft function between the two groups was not statistically significant. From this we can assume that delayed graft function is in DKT is similar to SKT.

DKT theoretically poses greater immunological challenge by providing more nephron mass to activate the immune system. However, acute rejection occurred in 12–20.8% in patients with DKT as compared to 17.6%–34.3% in SKT [16, 18, 19, 22, 24]. In most of these studies the occurrence of acute rejection in DKT was not significant statistically. The reason for less rejection despite increased nephron mass could be due the facts that recipient of DKT is the elderly who have blunted immune responses.

Since the first report of DKT by Johnson et al. [42] for graft survival, multiple comparative studies have been published on patient and graft survival in DKT and SKT. Various studies assessed graft survival at various intervals and it was found to be comparable with SKT. Johnson et al. and Remuzzi et al. reported 100% graft survival at 6-month follow-up [15, 16] in patients with DKT. Graft survival at 1 year has been reported to be 87–96% in various studies [36, 42–44]. Some studies have reported graft survival at 2 and 3 years as 96% and 93%, respectively [24, 44]. All these studies reported similar graft survival for both DKT and SKT except Jerius et al. [33] who reported better 1- and 2-year graft survival in DKT (96/96%) compared to SKT (77/73%). Gill and his colleague [18] found that death-censored allograft survival of DKT and extended criteria donor transplants were not significantly different up to 4 years after transplant. Snanoudj et al. [24] found that Kaplan–Meier estimates of non-death-censored graft survival up to 3 years were similar between DKT and SKT.

Patient survival is another important outcome and has been reported by various authors at various intervals. Six-month patient's survival has been 100% in various studies [15, 16]. Similarly 1-year survival has been reported as 96–98% [36, 42]. Lu et al. [20] followed their patients for 2 years and reported patient survival as 86% by the end of two years. Snanoudj et al. [24] found that Kaplan–Meier estimates of patient survival were similar up to 3 years in both DKT and SKT. Five-year graft survival has been reported as 87.3% in one study [19].  Table 4 summarizes the outcome of DKT reported in various studies.

These findings suggest that graft and patient survival in DKT is encouraging and comparable with SKT. Keeping in view similar surgical complications risk and similar incidence of delayed graft function and rejection with reasonable survival benefit DKT is considered as one of the viable option. In 2014 around 2,885 (17%) kidneys were discarded in USA [45]. Discard rate in Europe though 7.5% (304 donors) is still high [3]. Discard of 7.5–17% kidneys across the globe is an alarming figure. One can significantly reduce discard of these precious kidneys by implanting them through DKT. For instant, reducing discard rate in USA by 50% will provide around 1480 kidneys which can be utilized for DKT. Therefore, instead of discarding ECD kidney, one can sensibly allocate them for DKT. This will provide chronic kidney disease patients with an opportunity to come off dialysis and lead a healthy life with full functional status.

## 7. New Prospects and Future Direction

With the abundance of evidence based literature and cumulative experience now available for DKT, the transplant community has continued to open new frontiers for DKT. The preferred surgical technique in many developed centers has shifted from open donor nephrectomy to laparoscopic hand assisted nephrectomy. Another modern alternative is robotic assisted surgery which was first reported by Frongia et al. 2013 for dual kidney implantation [46]. The procedure was carried out by a 7-port intraperitoneal approach using the da Vinci surgical system. The total operative time was 400 minutes and blood loss was 120 ml. There were no intraoperative complications. The patient was discharged on the seventh postoperative day with normal renal function. They concluded that minimally invasive robotic assisted technology is a promising technique that provides exceptional patient outcomes by reducing operative morbidity, immobilization, and time to recovery, while affording better esthetic results. Further experience is required for robotic assisted surgery for dual kidney implantation.

Most patients with cirrhotic liver have either preexisting chronic kidney disease or develop acute kidney injury which results in chronic kidney disease. Combined liver and kidney transplant is becoming increasingly common. Di Laudo et al. [47] reported their experience of combined liver and DKT in 2016 and found no difference in graft and patient survival outcome with combined liver and SKT.

## 8. Conclusion

DKT is helpful in expanding donor pool and preventing discard. Various histological and clinical parameters are used to select a donor. There is a need to integrate histological score into multifactor score and to develop a consensus in selection of the donor for DKT. Recent advances and experience have accorded the use of various surgical techniques without compromising the rates of surgical complications. Long term graft and patient survival are promising and comparable to SKT.

## Figures and Tables

**Table 1:** Selection criteria for the donors.

Authors	Journal name/year	Number/surgical technique/immunosuppression used/selection criteria	Outcome
Johnson et al. [15]	Journal of Surgery/1996	9/Dual kidneys were transplanted intraperitoneally or through bilateral extraperitoneal incision/induction was either with ATG^*∗*^ or OKT3^*∗*^ followed by cyclosporine, AZA and prednisolone/selection Criteria: donor older than 60 years and/or long history of hypertension or diabetes with cold ischemia time less than 30 hours. Further criteria included creatinine clearance levels between 80 and 40 mL/min and with kidneys that showed less than 40% glomerulosclerosis without severe interstitial fibrosis or arteriosclerosis on biopsy	100% graft survival at 6 months

Remuzzi et al. [16]	Journal of American Society of Nephrology/1999	24/Bilateral placement through double inguinal incision/Prednisolone, Cyclosporine & mycophenolate mofetil. No comment on induction/Selection Criteria: brain dead donors having 1 of the following• Age > 60 year• History of diabetes or hypertension• Clinical proteinuria (urinary protein excretion rate up to 3 g/24 h) plus renal histopathology score of 4–6	100% graft survival at 6 months and 93% at 3 years

Lu et al. [20]	Archives of Surgery/1999	50/Bilateral placement in right and left iliac fossa via midline extra peritoneal approach/Cyclosporine, steroids & MMF^*∗*^ or AZA^*∗*^. Induction with OKT3^*∗*^ or IL-2^*∗*^ inhibitor/Selection Criteria: ECD^*∗*^ refused by other centers due to hypertension, donor instability, donor age or pretransplantation biopsy result and creatinine clearance less than 90 ml/minute in donors with age greater than 60 years	86% graft survival at 1 year and 76% at 2 years

Andrés et al. [17]	Transplantation/2000	21/Each kidney was implanted extraperitoneally in each iliac fossae/Prednisolone, cyclosporine or tacrolimus and MMF/selection criteria/age greater than 75 or 60–74 and pregraft biopsy showing greater than 15% glomerulosclerosis and lesser than 50% glomerulosclerosis	95% graft survival at 1 year and 93% at 2 years

Gill et al. [18]	Transplantation/2008	625 received DKT/no comment on surgical technique or immunosuppression/selection criteria: any 2 of the following criteria present: age greater than 60 years, creatinine clearance greater than 65 mL/min, rising serum creatinine greater than 2.5 mg/dL at retrieval, chronic hypertension or type 2 diabetes mellitus, and glomerulosclerosis on biopsy between 15% and 50%	79.8% graft survival at 3 years

Navarro et al. [23]	Journal of Urology/2008	23 non heart beating DKT/ipsilateral dual transplant, the 2 kidneys from a single donor are implanted into the right iliac fossa with anastomoses to the common (right kidney) and external iliac (left kidney) arterial circulation/no comment on immunosuppression medications/selection criteria: machine perfusion pressure flow index 0.4 mL/min per 100 g/mm Hg and glutathione transferase was greater than 100 IU/L/100 grams renal mass	Glomerular filtration rate at 3 and 6 months was 46.2 & 45.5 ml/minute

Snanoudj et al. [24]	American Journal of Transplantation/2009	81/Allografts were placed either monolaterally or bilaterally, with one or two classical iliac incisions, respectively/received IL-2 receptor antagonist or ATG^*∗*^. Cyclosporine or tacrolimus, prednisolone and MMF were used after induction/selection criteria: ECD donors aged > 65 years and estimated glomerular filtration rate was between 30 and 60 mL/min	Glomerular filtration rate at 12 months 47.8 mL/min

Ekser et al. [19]	American Journal of Transplantation/2010	100/Unilateral extraperitoneal placement via Gibson incision/Induction therapy consisted of antithymocyte globulin (ATG) or Basiliximab. Maintenance immunosuppressive sirolimus or everolimus either without a calcineurin inhibitor (CNI^*∗*^) or with a reduced CNI/selection criteria: high risk marginal donor (donor with age ≥ 70 0r 60–69 with either one of the Serum creatinine > 1.5 mg/dl, calculated creatinine clearance ≤ 60 ml/minute, history of hypertension and or diabetes, proteinuria > than 1 gram, cause of death cerebrovascular accident) plus biopsy score 4–6	Glomerular filtration rate at 1 year and 2 years was 115 ± 32 and 128 ± 45 ml/minute

Klair et al. [28]	American Journal of Transplantation/2013	1308/No comment on surgical technique or immunosuppressive medications used/selection criteria: DKT was done in KDRI score 1.4, 1.41–1.8, 1.8–2.2 and greater than 2. DKT showed superior graft survival when KDRI was greater than 2	Five-year graft survival rates of SKT and DKT by KDRI were as follows: 1.4 (74%, 72%), 1.41–1.8 (63%, 64%), 1.81–2.2 (55%, 59%) and >2.2 (48%, 54%).

Frutos et al. [21]	Nefrolgia/2012	20/Dual kidney transplantation was performed through 2 independent incisions in each of the recipient's iliac fossae/Immunosuppressive medications included induction with basiliximab. Maintenance immunosuppression included prednisolone, tacrolimus & MMF/selection criteria: DKT was done through biopsy scoring plus clinical parameter including Donor's age, medical history, kidney size and creatinine clearance were also considered	Creatinine clearance at 6 months and 1 year was 59.0 ± 18 ml/minute 55.0 ± 18.5 ml/minute

Islam et al. [22]	Journal of Transplantation/2016	29/Extraperitoneal placement in right iliac fossa through curvilinear incision/high risk recipients received ATG and the rest either daclizumab or basiliximab. Maintenance immunosuppression consisted of tacrolimus, MMF, and prednisone/selection criteria: Expanded criteria donors (ECD), defined as deceased donors (1) greater than 60 years old or (2) greater than 50 years old and with at least 2 of the following criteria: (a) a history of hypertension, (b) terminal serum creatinine greater than 1.5 mg/dL, or (c)death due to a cerebrovascular accident OR Kidneys from standard criteria donors (SCD) that were deemed functionally compromised due to high serum creatinine, poor pump characteristics, or unfavorable histology on biopsy	Median e GFR^*∗*^ (IQR) in mL/min/1.73 m^2^ at 12 months was 56.0 (42.6–67.9) & at 24 months 53.4 (46.4–66.4) ml/minute

^*∗*^. MMF^*∗*^ (mycophenolate mofetil), AZA^*∗*^ (azathioprine), IL-2^*∗*^ (interleukin-2), ECD^*∗*^ (extended Criteria Donor), e GFR^*∗*^ (estimated glomerulofilteration rate), ATG^*∗*^ (antithymocyte globulin), and SCD^*∗*^ (standard criteria donor), OKT3^*∗*^ (muromonab-CD3).

**Table 2:** Recipient characteristics DKT versus SKT.

Author	Journal/year/number of recipient	DKT					SKT			
		Age	Immunosuppression	Weight	BMI	Comorbid	Age (years)	Weight	BMI	Comorbid
Remuzzi et al. [16]	Journal of American Society of Nephrology/1999/24 DKT	59.4 ± 9.9	Prednisolone, Cyclosporine & mycophenolate mofetil. No comment on induction	71.4 ± 19.1	25.3 ± 5.4	HTN^*∗*^ 77.3% DM^*∗*^ 4.4%	50.2 ± 12.1	73.1 ± 16.2	25.3 ± 4.7	HTN^*∗*^ 71.7% DM^*∗*^ 6.4%

Lu et al. [20]	Archives of Surgery/1999/50 DKT	57 ± 11	Cyclosporine, steroids & MMF^*∗*^ or AZA^*∗*^. Induction with OKT3^*∗*^ or IL-2^*∗*^ inhibitor	—	—	—	50 ± 12	—	—	—

Snanoudj et al. [24]	American Journal of Transplantation/2009/81 DKT	69.4 ± 3.0	Received IL-2 receptor antagonist or ATG^*∗*^. Cyclosporine or tacrolimus, prednisolone and MMF were used after induction	68.4 ± 14.1	24.3 ± 4.1	DM^*∗*^ 18.5% ischemic cardiopathy 18.2%	59.9 ± 6.3	72.8 ± 17.0	25.1 ± 4.7	DM^*∗*^ 18.6% Ischemic Cardiopathy 12.9%

Ekser et al. [19]	American Journal of Transplantation/2010/100 DKT	61.7 ± 5.6	Induction therapy consisted of antithymocyte globulin (ATG) or Basiliximab. Maintenance immunosuppressive sirolimus or everolimus either without a calcineurin inhibitor (CNI^*∗*^) or with a reduced CNI	—	25.5 ± 3.5		57.7 ± 8.6	—	24.5 ± 3.4	—

Klair et al. [28]	American Journal of Transplantation/2013/1308 DKT	58.9 ±10.5	No comment on immunosuppressive medications used	—	25.1	DM^*∗*^ 37.5% HTN^*∗*^ 25.6%	49.6 ± 14.8	—	29.6	DM^*∗*^ 28.4% HTN^*∗*^ 34.9%

Bunnapradist et al. [31]	Journal of American Society of Nephrology/403 DKT	55.1 ± 11.5	No comment on immunosuppressive medications used	77.1 ± 17.1	—	—	48.1 ± 13.7	77.4 ± 19.2	—	—

^*∗*^. HTN^*∗*^ (hypertension), DM^*∗*^ (diabetes mellitus), PRA^*∗*^ (panel reactive antibody), HLA MM^*∗*^ (human leukocyte antigen mismatches), M^*∗*^ (male), F^*∗*^(female), MMF^*∗*^ (mycophenolate mofetil), AZA^*∗*^(azathioprine), IL-2^*∗*^ (interleukin-2), ATG^*∗*^ (antithymocyte globulin), and OKT3^*∗*^ (muromonab-CD3).

**Table 3:** Surgical complication of DKT versus SKT.

Author	Journal/year	DKT		SKT	Significance
		Surgical technique/number/immunosuppression	Complication	Complications	*P* value
Frutos et al. [21]	Nefrologia/2012	20 bilateral Kidney placement through 2 independent incisions in each of the recipient's iliac fossae/induction with basiliximab + prednisolone, tacrolimus & MMF^*∗*^	Hemorrhage 8 (40%)	Hemorrhage 10 (25%)	NSN
			Lymphocele 3 (15%)	Lymphocele 2 (5%)	SNS
			Resurgery 1 (5%)	Resurgery 1 (2.5%)	NS
			Arterial thrombosis 2 (10%)	Arterial thrombosis 2 (5%)	

Snanoudj et al. [24]	American Journal of Transplantation/2009	81 monolateral or bilateral placement with one or two classical iliac incisions/received IL-2^*∗*^ receptor antagonist or ATG^*∗*^. Cyclosporine or tacrolimus, prednisolone and MMF^*∗*^ were used after induction	Eventration, parietal abscess 6 (7.4%),	Eventration, parietal abscess 8 (11.4%)	NS
			Ureteral stenosis 9 (11.1%),	Ureteral stenosis 12 (17.1%)	NSN
			Urinary fistula 9 (11.1%)	Urinary fistula 15 (21.4%)	SNS
			Graft artery stenosis 9 (11.1%),	Graft artery stenosis 3 (4.3%)	NSN
			Graft partial infarction 3 (3.7%),	Graft partial infarction 4 (5.7%)	SNS
			Artery thrombosis 5 (6.2%) &	Artery thrombosis 2 (2.9%)	NS
			Vein thrombosis 6 (7.4%)	Vein thrombosis 1 (1.4%)	
			Hemorrhage 10 (12.3%)	Hemorrhage 9 (12.9%)	

Remuzzi et al. [16]	Journal of American Society of Nephrology/1999	24 bilateral placement through double inguinal incision/Prednisolone, Cyclosporine & mycophenolate mofetil	Urinary tract fistula 4	Urinary tract fistula 1	—
			Sepsis from urinary Tract 2	Sepsis from urinary Tract 2	—
			Deep vein thrombosis 1	Deep vein thrombosis 1	—
			Hematoma 1	Hematoma 1	—
			Gastrointestinal Bleeding 1	Gastrointestinal Bleeding 0	—
			Bowel occlusion 0	Bowel occlusion 0	—

Ekser et al. [19]	American Journal of Transplantation/2010	100 unilateral extraperitoneal placement via Gibson incision/Induction therapy consisted of antithymocyte globulin (ATG) or Basiliximab. Maintenance immunosuppressive sirolimus or everolimus either without a calcineurin inhibitor (CNI^*∗*^) or with a reduced CNI^*∗*^ dosage	*Renal vein thrombosis 1 (1%)*	*Renal vein thrombosis 1 (1.4%) *	NS
			*Wound dehiscence 5 (5%)*	*Wound dehiscence 2 (2.7%)*	NSN
			*Lymphocele 3 (3%)*	*Lymphocele 2 (2.7%)*	SNS
			*Hematoma 1 (1%)*	*Hematoma 0 (0%)*	NS
			*Incisional hernia 1 (1%)*	*Incisional hernia 0 (0%)*	
			*Stenosis of ureteroneocystoanastomoses 2 (2%)*	*Stenosis of ureteroneocystoanastomoses 2 (2.7%)*	NS

Islam et al. [22]	Journal of Transplantation/2016	29 extraperitoneal placement in right iliac fossa through curvilinear incision/high risk recipients received ATG^*∗*^ and the rest either daclizumab or basiliximab. Maintenance immunosuppression consisted of tacrolimus, MMF^*∗*^, and prednisone	Urologic complications 4/29 (14%)	Urologic complications 10/487 (2%)	S
			All 4 having ureteral stricture	6 out of 10 have anastomotic strictures and 4 has urine leak	

^*∗*^. IL-2^*∗*^ (interleukin-2), ATG^*∗*^ (antithymocyte globulin), MMF^*∗*^ (mycophenolate mofetil), and CNI (calcineurin inhibitors).

**Table 4:** Outcome of DKT.

Author	Journal/year/number Of DKT	Number and surgical technique/Immunosuppression for DKT	Delayed graft function		Acute rejection		Graft survival/kidney function (creatinine or GFR)		Patient survival	
			DKT	SKT	DKT	SKT	DKT	SKT	DKT	SKT
Johnson et al. [42]	Transplantation/1996 Surgery/1996	Six paired kidneys were placed intraperitoneally, while the remaining four pairs were placed in bilateral retroperitoneal iliac fossa locations/no comment on immunosuppressive medications	—	—	—	—	Overall graft survival of 90.0% and actuarial 1-year graft survival of 83.3%. (no death occurred in cohort)	—	—	—
Johnson et al. [15]		Dual kidneys were transplanted intraperitoneally or through bilateral extraperitoneal incision/Induction was either with ATG^*∗*^ or OKT3^*∗*^ followed by cyclosporine, AZA and prednisolone	—	—	—	—	Graft survival at 6 month in dual was 100% (no death till 6 month) in <50 years cadaveric kidney donor and 75% graft t survival (no comment whether death censored or not) in recipient who got kidney from cadaveric donor age > 60 years	62.5 ± 5.4 & 24.5 ± 5.3 ml/minute in age < 50 & age > 60. Graft survival 95% in age < 50 & 75% survival in age > 60 years	100% in recipient of cadaveric kidneys from less than 50 year & 83% from donor greater than 60 years	95% & 83% at 6 months in age < 50 & age > 60 years

Alfrey et al. [43]	Transplantation/1997	20 DKT as two single kidneys on the back table. Through a midline incision the iliac vessels were exposed via extraperitoneal dissection/all patients received cyclosporine-based triple-drug therapy	9%	45%			87% survival at 1 year (nondeath censored. Graft loss defined as return to dialysis or death)	Cr 2.8 ± 2.0 mg/dl at 4 weeks & 81% survival at 1 year	Cr .4 ± 0.5 mg/dl at 4 weeks & 93% survival at 1 year	96% survival at 1 year

Stratta and Bennett [44]	Transplant Proc/1997	60 DKT (25 young donors 35 old donors)/No comments on surgical teqhnique or immunosuppressive medications used	—	—	—	—	90.8% 1 year survival (No comment whether death censored or not)	87.5% 1 year survival	—	—

Lu et al. [20]	Archives of Surgery/1999	50 bilateral placement in right and left iliac fossa via midline extra peritoneal approach/Cyclosporine, steroids & MMF or AZA. Induction with OKT3 or IL-2 inhibitor	26%	39%	0.2 ± 0.5	0.7 ± 0.9	Death censored graft survival at 2 years was 85%	Death censored graft survival at 2 years was 84%. years in ECD SKT and 86% in control single	86% 2-year survival	96% 2-year survival

Remuzzi et al. [16]	Journal of American Society of Nephrology/1999	24 bilateral placement through double inguinal incision/Prednisolone, Cyclosporine & mycophenolate mofetil. No comment on induction	20.8%	20.8%	20.8%	18.8%	Cr 1.5 ± 0.4 mg/dl & 100% Survival at 6 months (no death till follow-up)	Cr 1.9 ± 0.7 mg/dl 100% Survival at 6 months	100% Survival at 6 months	100% survival at 6 months

Jerius et al. [33]	Journal of Urology/2000	28 kidneys were placed bilaterally or unilaterally using standard right and left lower quadrant extraperitoneal approaches/4 patients receiving pancreas received OKT3 induction. Triple drug immunosuppression consisted of cyclosporine or tacrolimus, azathioprine or mycophenolate mofetil and prednisone was used	6 out 28 cases	7 out of 31 cases	—	—	In an intent to treat analysis with inclusion of all patients 1 and 2-year graft survival rates were 93% and 86%. These differences were not statistically significant. The data were then adjusted to eliminate nongraft dependent loss factors. The patient in each group with loss due to subacute humoral rejection was excluded from analysis. The graft loss due to patient noncompliance in group 1 was treated as censored data at the time of loss instead of graft failure. With these adjustments 1- and 2-year graft survival rates for DKT were 96% and 96% which was significantly better than SKT	1- and 2-year graft survival rates of 77% and 73%	—	—

Lee et al. [32]	Journal of American College of Physician/1999	41 dual kidneys were procured in the usual fashion and were prepared as 2 single kidneys on the back table. Through a midline incision the iliac vessels were exposed by extraperitoneal dissection; one kidney was anastomosed to the left iliac vessels, and the other to the right iliac vessels/the majority of the recipients transplanted received cyclosporine based triple therapy that included mycophenolate mofetil and prednisone	24%	33%	—	—	GFR at 1 year 54 ± 23 ml/min & 1 year graft survival 89% (graft loss defined as permanent return to dialysis)	GFR at 1 year 57 ± 25 ml/min 1-year graft survival 90%	1-year patient survival 97%	1-year patient survival 98%

Gill et al. [18]	Transplantation/2008	625 received DKT/no comment on surgical technique or immunosuppression	29.3%	33.6% ECD^*∗*^	12.1% at 1 year	17.6% at 1 year	Death-censored allograft survival of DKT and ECD transplants were not significantly different up to 4 years after transplant	Death-censored allograft survival of DKT and ECD transplants were not significantly different up to 4 years after transplant	—	—

Snanoudj et al. [24]	American Journal of Transplantation/2009	81 monolateral or bilateral placement with one or two classical iliac incisions/received IL-2^*∗*^ receptor antagonist or ATG^*∗*^. Cyclosporine or tacrolimus, prednisolone and MMF^*∗*^ were used after induction	31.6%	51.4% (Significant)	12.3% at 1 year	34.3% at 1 year	Kaplan–Meier estimates of non-death-censored graft survival up to 3 years similar	Kaplan–Meier estimates of non- death-censored graft survival up to 3 years similar	Kaplan–Meier estimates of patient survival similar up to 3 years	Kaplan–Meier estimates of patient survival similar up to 3 years

Frutos et al. [21]	Nefrologia/2012	20 bilateral Kidney placement through 2 independent incisions in each of the recipient's iliac fossae/induction with basiliximab + prednisolone, tacrolimus & MMF^*∗*^	30%	35%	—	—	GFR at 1 year 55.0 ± 18.5. Graft survival at 3 years was 90% (not death censored).	GFR at 1 year 51.3 ± 6.2	—	—

Ekser et al. [19]	American Journal of Transplantation/2010	100 unilateral extraperitoneal placement via Gibson incision/Induction therapy consisted of antithymocyte globulin (ATG^*∗*^) or basiliximab. Maintenance immunosuppressive sirolimus or everolimus either without a calcineurin inhibitor (CNI^*∗*^) or with a reduced CNI^*∗*^	31%	30%	17%	28%	Actuarial Kaplan–Meier graft survival curves at 5-year follow-up was 90.9% (no comment about death censored or not death censored)	4 GFR at 5 year 9 ± 13 (12 patients)	5-year patient survival, 95.6%	5-year patient survival, 87.3%

Islam et al. [22]	Journal of Transplantation/2016	29 extraperitoneal placement in right iliac fossa through curvilinear incision/high risk recipients received ATG and the rest either daclizumab or basiliximab. Maintenance immunosuppression consisted of tacrolimus, MMF, and prednisone	10.3%	9.2%	20.7%	22.4%	Median e GFR (IQR) at 36 months 45.9 ml/minute (36.8–62.6). Actuarial graft survivals 93% at 3 years (No death occurred in this cohort)	Median e GFR (IQR) at 36 months 56.7 (43.7–71.8)	Actuarial patient survivals 100% at 3 years	

^*∗*^. IL-2^*∗*^ (interleukin-2), ATG^*∗*^ (antithymocyte globulin), MMF^*∗*^ (mycophenolate mofetil), AZA^*∗*^ (azathiopurine), CNI^*∗*^ (calcineurin inhibitors), ECD^*∗*^ (extended criteria donor), e GFR^*∗*^ (estimated glomerulofilteration rate), and GFR^*∗*^ (glomerulofilteration rate).
